# [μ-Bis(di-*o*-tolyl­phosphan­yl)methane-1:2κ^2^ *P*:*P*′]deca­carbonyl-1κ^3^ *C*,2κ^3^ *C*,3κ^4^ *C*-*triangulo*-triruthenium(0)–methanol (8/1)

**DOI:** 10.1107/S1600536811000791

**Published:** 2011-01-15

**Authors:** Omar bin Shawkataly, Imthyaz Ahmed Khan, H. A. Hafiz Malik, Chin Sing Yeap, Hoong-Kun Fun

**Affiliations:** a Chemical Sciences Programme, School of Distance Education, Universiti Sains Malaysia, 11800 USM, Penang, Malaysia; b X-ray Crystallography Unit, School of Physics, Universiti Sains Malaysia, 11800 USM, Penang, Malaysia

## Abstract

The asymmetric unit of the title compound, [Ru_3_(C_29_H_30_P_2_)(CO)_10_].1/8CH_3_OH, contains two *triangulo*-triruthenium mol­ecules with similar configurations and a methanol solvent mol­ecule (fractional site occupancy for the latter = 0.25). The bis­(di-*o*-tolyl­phosphan­yl)methane ligand bridges an Ru—Ru bond and its P atoms are equatorial with respect to the Ru_3_ triangle. The phosphine-substituted Ru atoms each bear one equatorial and two axial terminal carbonyl ligands whereas the unsubstituted Ru atom carries two equatorial and two axial terminal carbonyl ligands. The dihedral angles between the two benzene rings attached to each P atom are 76.26 (13) and 74.76 (15)° for the first mol­ecule and 77.21 (13) and 75.68 (14)° for the second. In the crystal, mol­ecules are linked into [001] chains *via* inter­molecular C—H⋯O hydrogen bonds. Weak inter­molecular C—H⋯π inter­actions also occur.

## Related literature

For general background to *triangulo*-triruthenium derivatives, see: Bruce, *et al.* (1985, ▶ 1988*a* ▶,*b* ▶). For related structures, see: Shawkataly *et al.* (1998 ▶, 2004 ▶, 2010 ▶). For the synthesis of bis­(di-*o*-tolyl­phosphan­yl)methane, see: Filby *et al.* (2006 ▶). For the stability of the temperature controller used in the data collection, see: Cosier & Glazer (1986 ▶).
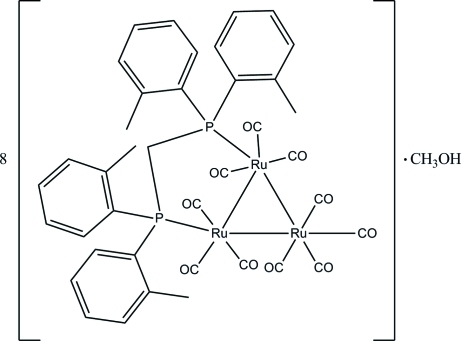


## Experimental

### 

#### Crystal data

8[Ru_3_(C_29_H_30_P_2_)(CO)_10_]·CH_4_O
*M* *_r_* = 8222.28
Monoclinic, 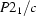

*a* = 22.7156 (13) Å
*b* = 19.9023 (11) Å
*c* = 18.4979 (10) Å
β = 106.561 (1)°
*V* = 8015.8 (8) Å^3^
*Z* = 1
Mo *K*α radiation
μ = 1.25 mm^−1^
*T* = 100 K
0.23 × 0.10 × 0.06 mm

#### Data collection

Bruker APEXII DUO CCD diffractometer
Absorption correction: multi-scan (*SADABS*; Bruker, 2009 ▶) *T*_min_ = 0.758, *T*_max_ = 0.932
107136 measured reflections
28473 independent reflections
20091 reflections with *I* > 2σ(*I*)
*R*_int_ = 0.056

#### Refinement

*R*[*F* ^2^ > 2σ(*F* ^2^)] = 0.035
*wR*(*F* ^2^) = 0.082
*S* = 1.03
28473 reflections
990 parameters
1 restraint
H-atom parameters constrained
Δρ_max_ = 1.14 e Å^−3^
Δρ_min_ = −0.72 e Å^−3^

### 

Data collection: *APEX2* (Bruker, 2009 ▶); cell refinement: *SAINT* (Bruker, 2009 ▶); data reduction: *SAINT*; program(s) used to solve structure: *SHELXTL* (Sheldrick, 2008 ▶); program(s) used to refine structure: *SHELXTL*; molecular graphics: *SHELXTL*; software used to prepare material for publication: *SHELXTL* and *PLATON* (Spek, 2009 ▶).

## Supplementary Material

Crystal structure: contains datablocks global, I. DOI: 10.1107/S1600536811000791/hb5784sup1.cif

Structure factors: contains datablocks I. DOI: 10.1107/S1600536811000791/hb5784Isup2.hkl

Additional supplementary materials:  crystallographic information; 3D view; checkCIF report

## Figures and Tables

**Table 1:** Selected bond lengths (Å)

Ru1*A*—P1*A*	2.3451 (7)
Ru2*A*—P2*A*	2.3422 (7)
Ru1*B*—P1*B*	2.3450 (7)
Ru2*B*—P2*B*	2.3459 (7)

**Table 2:** Hydrogen-bond geometry (Å, °) *Cg*1 is centroid of the C14*A*–C19*A* benzene ring.

*D*—H⋯*A*	*D*—H	H⋯*A*	*D*⋯*A*	*D*—H⋯*A*
C11*B*—H11*B*⋯O1*B*^i^	0.93	2.54	3.251 (3)	134
C24*A*—H24*A*⋯O5*A*^ii^	0.93	2.49	3.240 (4)	138
C29*A*—H29*B*⋯*Cg*1	0.96	2.96	3.702 (4)	135

Symmetry codes: (i) 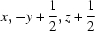; (ii) 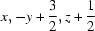.

**Fig. 1.**
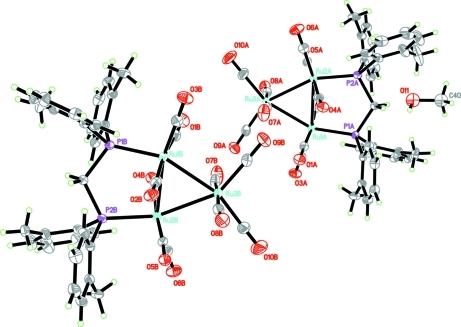
The molecular structure of the title compound with 50% probability ellipsoids for non-H atoms.

**Fig. 2.**
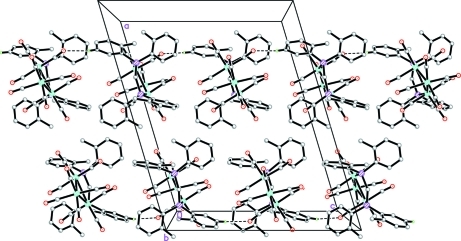
The crystal packing of the title compound, viewed down the a axis, showing the molecules linked into chains along c axis. Hydrogen atoms not involved in the hydrogen-bonding (dashed lines) and solvent molecules have been omitted for clarity.

## supplementary crystallographic information

### Comment

A large number of substituted derivatives, Ru_3_(CO)_12-n_*L*_n_ (*L* = group 15 ligand) have been reported (Bruce *et al.*, 1985, 1988*a*,*b*; Bruce *et al.*, 1985). As part of our study on the substitution of transition metal-carbonyl clusters with mixed-ligand complexes, we have published several structures of *triangulo*-triruthenium-carbonyl clusters containing mixed P/As and P/Sb ligands (Shawkataly *et al.*, 1998, 2004, 2010). Herein we report the synthesis and structure of the title compound.

The asymmetry unit of title compound consists of two molecule of *triangulo*-triruthenium complex (*A* & *B*) and one-quater molecule of methanol solvent (Fig. 1). The bis(di-*o*-tolylphosphanyl)methane ligand bridges the Ru1–Ru2 bond. The phosphine ligand is equatorial with respect to the Ru triangle. The Ru1 and Ru2 atoms each carries one equatorial and two axial terminal carbonyl ligands whereas the Ru3 atom carries two equatorial and two axial terminal carbonyl ligands. The dihedral angles between the two benzene rings (C1–C6/C7–C12 and C14–C19/C20–C25) are 76.26 (13) and 74.76 (15)° in molecule *A* for the two diphenylphosphanyl groups respectively whereas these angles are 77.21 (13) and 75.68 (14)° in molecule *B*.

In the crystal, the molecules are linked into one-dimensional chains along *c* axis *via* intermolecular C11B—H11B···O1B and C24A—H24A···O5A hydrogen bonds (Fig. 2, Table 1). Weak intermolecular C—H···π interactions (Table 1) consolidate the packing.

### Experimental

All manipulations were performed under a dry oxygen-free nitrogen atmosphere using standard Schlenk techniques. All solvents were dried over sodium and distilled from sodium benzophenone ketyl under dry oxygen free nitrogen. Bis(di-*o*-tolylphosphanyl)methane (Filby *et al.*, 2006) was prepared by reported procedure. Equimolar quantities of Ru_3_(CO)_12_ and bis(di-*o*-tolylphosphanyl)methane were stirred in THF (25 ml) under nitrogen. About 0.2 ml of diphenylketyl radical anion initiator was introduced into the reaction mixture under a current of nitrogen. The reaction mixture turned intense red. After 10 minutes of stirring the solvent was removed under vacuum. The reaction mixture was separated by TLC (acetone: hexane, 10:90) three bands appeared. The major band (red) was separated and characterized. Orange needles of the title compound were grown by slow solvent / solvent diffusion of CH_3_OH into CH_2_Cl_2_.

### Refinement

All hydrogen atoms were positioned geometrically and refined using a riding model with C—H = 0.93–0.97 Å and *U*_iso_(H) = 1.2 or 1.5 *U*_eq_(C). Rotating group model was applied for the methyl group. The solvent molecule is modeled to be a methanol. The occupancy of methanol molecule is fixed to 0.25 and the O–C bond distance is restrained with a distance of 1.50 (1) Å. The methanol solvent is refined isotropically. The maximum and minimum residual electron density peaks of 1.14 and -0.72 e Å^-3^ were located 0.64 and 0.71 Å from the H40C and Ru2B atoms, respectively.

### Crystal data

**Table:**

8[Ru_3_(C_29_H_30_P_2_)(CO)_10_]·CH_4_O	*F*(000) = 4066
*M**_r_* = 8222.28	*D*_x_ = 1.703 Mg m^−^^3^
Monoclinic, *P*2_1_/*c*	Mo *K*α radiation, λ = 0.71073 Å
Hall symbol: -P 2ybc	Cell parameters from 9981 reflections
*a* = 22.7156 (13) Å	θ = 2.5–31.9°
*b* = 19.9023 (11) Å	µ = 1.25 mm^−^^1^
*c* = 18.4979 (10) Å	*T* = 100 K
β = 106.561 (1)°	Needle, orange
*V* = 8015.8 (8) Å^3^	0.23 × 0.10 × 0.06 mm
*Z* = 1	

### Data collection

**Table:**

Bruker APEXII DUO CCD diffractometer	28473 independent reflections
Radiation source: fine-focus sealed tube	20091 reflections with *I* > 2σ(*I*)
graphite	*R*_int_ = 0.056
φ and ω scans	θ_max_ = 32.4°, θ_min_ = 1.9°
Absorption correction: multi-scan (*SADABS*; Bruker, 2009)	*h* = −34→34
*T*_min_ = 0.758, *T*_max_ = 0.932	*k* = −26→29
107136 measured reflections	*l* = −25→27

### Refinement

**Table:**

Refinement on *F*^2^	Primary atom site location: structure-invariant direct methods
Least-squares matrix: full	Secondary atom site location: difference Fourier map
*R*[*F*^2^ > 2σ(*F*^2^)] = 0.035	Hydrogen site location: inferred from neighbouring sites
*wR*(*F*^2^) = 0.082	H-atom parameters constrained
*S* = 1.03	*w* = 1/[σ^2^(*F*_o_^2^) + (0.0264*P*)^2^ + 3.1179*P*] where *P* = (*F*_o_^2^ + 2*F*_c_^2^)/3
28473 reflections	(Δ/σ)_max_ = 0.002
990 parameters	Δρ_max_ = 1.14 e Å^−^^3^
1 restraint	Δρ_min_ = −0.72 e Å^−^^3^

### Special details

**Table:**

Experimental. The crystal was placed in the cold stream of an Oxford Cryosystems Cobra open-flow nitrogen cryostat (Cosier & Glazer, 1986) operating at 100.0 (1) K.
Geometry. All e.s.d.'s (except the e.s.d. in the dihedral angle between two l.s. planes) are estimated using the full covariance matrix. The cell e.s.d.'s are taken into account individually in the estimation of e.s.d.'s in distances, angles and torsion angles; correlations between e.s.d.'s in cell parameters are only used when they are defined by crystal symmetry. An approximate (isotropic) treatment of cell e.s.d.'s is used for estimating e.s.d.'s involving l.s. planes.
Refinement. Refinement of *F*^2^ against ALL reflections. The weighted *R*-factor *wR* and goodness of fit *S* are based on *F*^2^, conventional *R*-factors *R* are based on *F*, with *F* set to zero for negative *F*^2^. The threshold expression of *F*^2^ > σ(*F*^2^) is used only for calculating *R*-factors(gt) *etc*. and is not relevant to the choice of reflections for refinement. *R*-factors based on *F*^2^ are statistically about twice as large as those based on *F*, and *R*- factors based on ALL data will be even larger.

### Fractional atomic coordinates and isotropic or equivalent isotropic displacement parameters (Å^2^)

**Table:**

	*x*	*y*	*z*	*U*_iso_*/*U*_eq_	Occ. (<1)
Ru1A	0.220302 (9)	0.677332 (10)	0.085362 (11)	0.01552 (4)	
Ru2A	0.101706 (9)	0.679317 (10)	0.107074 (11)	0.01676 (4)	
Ru3A	0.155914 (10)	0.555256 (11)	0.087520 (12)	0.02137 (5)	
P1A	0.23685 (3)	0.79201 (3)	0.11282 (3)	0.01575 (12)	
P2A	0.09283 (3)	0.79598 (3)	0.09101 (3)	0.01690 (12)	
O1A	0.29531 (9)	0.64003 (11)	0.24630 (11)	0.0303 (4)	
O2A	0.14958 (9)	0.70117 (11)	−0.08085 (10)	0.0286 (4)	
O3A	0.33234 (9)	0.63612 (11)	0.03704 (11)	0.0343 (5)	
O4A	0.17311 (9)	0.69638 (10)	0.27390 (10)	0.0279 (4)	
O5A	0.02466 (9)	0.66139 (10)	−0.05763 (10)	0.0276 (4)	
O6A	−0.01057 (10)	0.63248 (12)	0.15217 (12)	0.0405 (6)	
O7A	0.18600 (13)	0.54704 (12)	0.25985 (12)	0.0469 (6)	
O8A	0.11455 (10)	0.55392 (11)	−0.08609 (12)	0.0376 (5)	
O9A	0.26875 (10)	0.47068 (11)	0.09090 (13)	0.0384 (5)	
O10A	0.05042 (12)	0.45816 (14)	0.07945 (16)	0.0622 (8)	
C1A	0.26433 (11)	0.84455 (13)	0.04703 (13)	0.0186 (5)	
C2A	0.27857 (12)	0.91336 (14)	0.05881 (15)	0.0241 (5)	
C3A	0.29300 (13)	0.94867 (15)	0.00094 (16)	0.0290 (6)	
H3AA	0.3026	0.9941	0.0078	0.035*	
C4A	0.29350 (13)	0.91861 (15)	−0.06618 (16)	0.0304 (6)	
H4AA	0.3021	0.9441	−0.1041	0.037*	
C5A	0.28134 (12)	0.85113 (15)	−0.07691 (15)	0.0261 (6)	
H5AA	0.2826	0.8303	−0.1214	0.031*	
C6A	0.26713 (11)	0.81470 (13)	−0.02028 (14)	0.0203 (5)	
H6AA	0.2592	0.7689	−0.0273	0.024*	
C7A	0.28948 (11)	0.80688 (12)	0.20754 (13)	0.0177 (5)	
C8A	0.35360 (12)	0.80422 (13)	0.22137 (14)	0.0206 (5)	
C9A	0.39014 (13)	0.81449 (14)	0.29506 (15)	0.0270 (6)	
H9AA	0.4326	0.8136	0.3046	0.032*	
C10A	0.36610 (14)	0.82584 (14)	0.35419 (15)	0.0303 (6)	
H10A	0.3920	0.8335	0.4024	0.036*	
C11A	0.30257 (14)	0.82582 (14)	0.34126 (15)	0.0277 (6)	
H11A	0.2856	0.8319	0.3810	0.033*	
C12A	0.26502 (13)	0.81657 (13)	0.26826 (14)	0.0233 (5)	
H12A	0.2226	0.8168	0.2595	0.028*	
C13A	0.16700 (11)	0.84055 (13)	0.11510 (15)	0.0207 (5)	
H13A	0.1742	0.8590	0.1654	0.025*	
H13B	0.1630	0.8782	0.0808	0.025*	
C14A	0.05547 (11)	0.82095 (12)	−0.00663 (13)	0.0184 (5)	
C15A	−0.00846 (12)	0.81429 (13)	−0.03756 (14)	0.0222 (5)	
C16A	−0.03425 (13)	0.83291 (14)	−0.11266 (15)	0.0270 (6)	
H16A	−0.0766	0.8298	−0.1333	0.032*	
C17A	0.00073 (14)	0.85565 (15)	−0.15703 (15)	0.0307 (6)	
H17A	−0.0179	0.8682	−0.2067	0.037*	
C18A	0.06415 (14)	0.85982 (15)	−0.12748 (16)	0.0305 (6)	
H18A	0.0883	0.8739	−0.1576	0.037*	
C19A	0.09110 (12)	0.84276 (14)	−0.05254 (15)	0.0232 (5)	
H19A	0.1335	0.8459	−0.0326	0.028*	
C20A	0.05500 (12)	0.84311 (14)	0.15128 (14)	0.0238 (5)	
C21A	0.03789 (15)	0.91107 (17)	0.14213 (18)	0.0368 (7)	
C22A	0.01195 (18)	0.9402 (2)	0.1944 (2)	0.0509 (10)	
H22A	−0.0012	0.9846	0.1876	0.061*	
C23A	0.00516 (15)	0.9050 (2)	0.25650 (19)	0.0448 (9)	
H23A	−0.0118	0.9260	0.2908	0.054*	
C24A	0.02358 (14)	0.83938 (18)	0.26684 (17)	0.0363 (7)	
H24A	0.0201	0.8156	0.3087	0.044*	
C25A	0.04761 (12)	0.80862 (16)	0.21368 (14)	0.0259 (6)	
H25A	0.0591	0.7637	0.2201	0.031*	
C26A	0.28218 (14)	0.95086 (14)	0.13111 (16)	0.0302 (6)	
H26A	0.2807	0.9983	0.1216	0.045*	
H26B	0.3200	0.9399	0.1683	0.045*	
H26C	0.2482	0.9382	0.1493	0.045*	
C27A	0.38495 (13)	0.79109 (16)	0.16094 (16)	0.0309 (6)	
H27A	0.4269	0.7783	0.1841	0.046*	
H27B	0.3839	0.8311	0.1316	0.046*	
H27C	0.3641	0.7555	0.1287	0.046*	
C28A	−0.05019 (12)	0.78985 (16)	0.00726 (16)	0.0307 (6)	
H28A	−0.0865	0.7706	−0.0263	0.046*	
H28B	−0.0614	0.8269	0.0338	0.046*	
H28C	−0.0292	0.7565	0.0428	0.046*	
C29A	0.0455 (2)	0.95446 (19)	0.0783 (2)	0.0667 (14)	
H29A	0.0342	0.9999	0.0855	0.100*	
H29B	0.0195	0.9378	0.0312	0.100*	
H29C	0.0875	0.9532	0.0774	0.100*	
C30A	0.26436 (12)	0.65347 (13)	0.18781 (15)	0.0217 (5)	
C31A	0.17239 (12)	0.69159 (13)	−0.01808 (15)	0.0215 (5)	
C32A	0.29057 (12)	0.65169 (14)	0.05549 (14)	0.0225 (5)	
C33A	0.14982 (12)	0.68880 (13)	0.21078 (15)	0.0216 (5)	
C34A	0.05662 (12)	0.66612 (13)	0.00228 (15)	0.0220 (5)	
C35A	0.03166 (13)	0.65093 (15)	0.13479 (15)	0.0256 (6)	
C36A	0.17545 (15)	0.55492 (15)	0.19671 (17)	0.0324 (6)	
C37A	0.13004 (13)	0.55914 (14)	−0.02258 (16)	0.0268 (6)	
C38A	0.22584 (14)	0.50192 (14)	0.08860 (16)	0.0277 (6)	
C39A	0.08977 (15)	0.49406 (16)	0.08308 (19)	0.0377 (7)	
Ru1B	0.282548 (9)	0.326339 (10)	0.936357 (11)	0.01576 (4)	
Ru2B	0.399429 (9)	0.309121 (10)	0.911319 (11)	0.01557 (4)	
Ru3B	0.354149 (10)	0.440860 (10)	0.922137 (12)	0.02002 (5)	
P1B	0.25992 (3)	0.21187 (3)	0.91452 (3)	0.01521 (12)	
P2B	0.40317 (3)	0.19375 (3)	0.93823 (3)	0.01592 (12)	
O1B	0.21008 (10)	0.36087 (11)	0.77383 (11)	0.0333 (5)	
O2B	0.35113 (9)	0.30992 (10)	1.10464 (10)	0.0276 (4)	
O3B	0.17110 (9)	0.37604 (11)	0.98056 (11)	0.0341 (5)	
O4B	0.32795 (9)	0.28896 (10)	0.74471 (10)	0.0260 (4)	
O5B	0.47536 (9)	0.33369 (10)	1.07514 (10)	0.0254 (4)	
O6B	0.51458 (9)	0.34127 (12)	0.86489 (12)	0.0348 (5)	
O7B	0.32373 (13)	0.43605 (11)	0.74972 (12)	0.0489 (7)	
O8B	0.39854 (10)	0.45298 (11)	1.09512 (11)	0.0333 (5)	
O9B	0.24943 (10)	0.53881 (11)	0.91616 (14)	0.0419 (6)	
O10B	0.46223 (11)	0.52956 (13)	0.91726 (16)	0.0551 (7)	
C1B	0.22076 (11)	0.19372 (12)	0.81543 (13)	0.0179 (5)	
C2B	0.15836 (11)	0.20918 (13)	0.78421 (14)	0.0204 (5)	
C3B	0.13122 (13)	0.19456 (14)	0.70791 (15)	0.0249 (6)	
H3BA	0.0896	0.2031	0.6869	0.030*	
C4B	0.16457 (15)	0.16782 (15)	0.66293 (16)	0.0312 (7)	
H4BA	0.1454	0.1586	0.6124	0.037*	
C5B	0.22658 (14)	0.15480 (15)	0.69321 (15)	0.0283 (6)	
H5BA	0.2495	0.1377	0.6630	0.034*	
C6B	0.25418 (12)	0.16751 (13)	0.76907 (15)	0.0225 (5)	
H6BA	0.2958	0.1584	0.7895	0.027*	
C7B	0.21822 (11)	0.16697 (12)	0.97280 (13)	0.0176 (5)	
C8B	0.19090 (12)	0.10265 (13)	0.95663 (14)	0.0212 (5)	
C9B	0.17052 (13)	0.07180 (14)	1.01206 (16)	0.0274 (6)	
H9BA	0.1519	0.0299	1.0019	0.033*	
C10B	0.17656 (13)	0.10076 (15)	1.08253 (16)	0.0286 (6)	
H10B	0.1630	0.0781	1.1187	0.034*	
C11B	0.20303 (12)	0.16365 (14)	1.09782 (15)	0.0243 (5)	
H11B	0.2076	0.1837	1.1445	0.029*	
C12B	0.22272 (11)	0.19645 (13)	1.04266 (14)	0.0196 (5)	
H12B	0.2394	0.2393	1.0525	0.023*	
C13B	0.32775 (11)	0.15592 (13)	0.93696 (15)	0.0206 (5)	
H13C	0.3194	0.1196	0.9005	0.025*	
H13D	0.3319	0.1362	0.9861	0.025*	
C14B	0.42883 (11)	0.13463 (13)	0.87695 (14)	0.0202 (5)	
C15B	0.43712 (12)	0.06521 (14)	0.89234 (15)	0.0250 (5)	
C16B	0.45645 (15)	0.02591 (15)	0.84122 (17)	0.0347 (7)	
H16B	0.4628	−0.0198	0.8509	0.042*	
C17B	0.46658 (17)	0.05181 (17)	0.77716 (18)	0.0422 (8)	
H17B	0.4794	0.0238	0.7443	0.051*	
C18B	0.45770 (15)	0.11917 (16)	0.76151 (16)	0.0339 (7)	
H18B	0.4637	0.1369	0.7176	0.041*	
C19B	0.43979 (12)	0.16037 (14)	0.81156 (14)	0.0242 (5)	
H19B	0.4349	0.2062	0.8016	0.029*	
C20B	0.45181 (11)	0.17630 (12)	1.03454 (13)	0.0176 (5)	
C21B	0.51592 (12)	0.17446 (13)	1.05094 (14)	0.0200 (5)	
C22B	0.55078 (13)	0.16466 (13)	1.12575 (15)	0.0245 (6)	
H22B	0.5933	0.1626	1.1367	0.029*	
C23B	0.52438 (14)	0.15791 (14)	1.18371 (16)	0.0293 (6)	
H23B	0.5488	0.1512	1.2329	0.035*	
C24B	0.46091 (15)	0.16130 (15)	1.16792 (15)	0.0303 (6)	
H24B	0.4425	0.1574	1.2066	0.036*	
C25B	0.42512 (13)	0.17053 (14)	1.09421 (15)	0.0242 (5)	
H25B	0.3826	0.1729	1.0840	0.029*	
C26B	0.11899 (12)	0.23870 (15)	0.82941 (15)	0.0276 (6)	
H26D	0.0837	0.2600	0.7960	0.041*	
H26E	0.1423	0.2713	0.8642	0.041*	
H26F	0.1059	0.2036	0.8569	0.041*	
C27B	0.18306 (14)	0.06476 (14)	0.88355 (15)	0.0279 (6)	
H27D	0.1715	0.0192	0.8896	0.042*	
H27E	0.2211	0.0652	0.8706	0.042*	
H27F	0.1516	0.0859	0.8441	0.042*	
C28B	0.42639 (13)	0.03133 (14)	0.96078 (16)	0.0283 (6)	
H28D	0.4298	−0.0165	0.9563	0.042*	
H28E	0.3861	0.0424	0.9640	0.042*	
H28F	0.4565	0.0466	1.0055	0.042*	
C29B	0.55012 (12)	0.18341 (15)	0.99213 (15)	0.0257 (6)	
H29D	0.5913	0.1980	1.0162	0.039*	
H29E	0.5295	0.2165	0.9559	0.039*	
H29F	0.5512	0.1414	0.9670	0.039*	
C30B	0.24043 (12)	0.34815 (13)	0.83281 (15)	0.0225 (5)	
C31B	0.32887 (11)	0.31609 (13)	1.04120 (14)	0.0200 (5)	
C32B	0.21339 (12)	0.35807 (14)	0.96398 (14)	0.0225 (5)	
C33B	0.35150 (12)	0.29737 (13)	0.80760 (15)	0.0207 (5)	
C34B	0.44434 (11)	0.32671 (12)	1.01505 (14)	0.0188 (5)	
C35B	0.47147 (12)	0.32800 (14)	0.88280 (15)	0.0229 (5)	
C36B	0.33376 (15)	0.43294 (14)	0.81318 (17)	0.0324 (7)	
C37B	0.38139 (12)	0.44392 (14)	1.03222 (16)	0.0247 (5)	
C38B	0.28929 (13)	0.50278 (14)	0.91896 (16)	0.0274 (6)	
C39B	0.42278 (14)	0.49617 (15)	0.91889 (18)	0.0324 (7)	
O11	0.1574 (5)	0.9239 (5)	0.2700 (6)	0.048 (2)*	0.25
H11O	0.1805	0.9126	0.3109	0.072*	0.25
C40	0.1575 (7)	0.9966 (5)	0.2638 (8)	0.045 (3)*	0.25
H40A	0.1651	1.0163	0.3130	0.067*	0.25
H40B	0.1184	1.0115	0.2324	0.067*	0.25
H40C	0.1892	1.0102	0.2418	0.067*	0.25

### Atomic displacement parameters (Å^2^)

**Table:**

	*U*^11^	*U*^22^	*U*^33^	*U*^12^	*U*^13^	*U*^23^
Ru1A	0.01660 (9)	0.01669 (9)	0.01359 (9)	−0.00121 (7)	0.00484 (7)	−0.00157 (7)
Ru2A	0.01694 (9)	0.01977 (10)	0.01422 (9)	−0.00106 (7)	0.00547 (7)	0.00060 (7)
Ru3A	0.02564 (11)	0.01627 (10)	0.02350 (11)	−0.00219 (8)	0.00913 (9)	0.00056 (8)
P1A	0.0161 (3)	0.0175 (3)	0.0128 (3)	−0.0016 (2)	0.0028 (2)	−0.0018 (2)
P2A	0.0163 (3)	0.0210 (3)	0.0134 (3)	0.0013 (2)	0.0041 (2)	−0.0005 (2)
O1A	0.0313 (11)	0.0381 (12)	0.0194 (10)	0.0038 (9)	0.0041 (8)	0.0050 (9)
O2A	0.0283 (11)	0.0374 (12)	0.0171 (9)	−0.0088 (9)	0.0016 (8)	0.0012 (8)
O3A	0.0257 (11)	0.0491 (14)	0.0309 (11)	0.0043 (9)	0.0124 (9)	−0.0054 (10)
O4A	0.0268 (10)	0.0366 (12)	0.0189 (10)	0.0024 (8)	0.0042 (8)	0.0006 (8)
O5A	0.0289 (10)	0.0339 (11)	0.0176 (9)	−0.0050 (8)	0.0028 (8)	−0.0040 (8)
O6A	0.0332 (12)	0.0565 (16)	0.0381 (13)	−0.0153 (10)	0.0203 (10)	−0.0029 (11)
O7A	0.0771 (19)	0.0372 (13)	0.0275 (12)	−0.0025 (12)	0.0167 (12)	0.0075 (10)
O8A	0.0439 (13)	0.0367 (13)	0.0293 (12)	−0.0007 (10)	0.0058 (10)	−0.0047 (9)
O9A	0.0382 (13)	0.0356 (13)	0.0440 (13)	0.0092 (10)	0.0159 (11)	0.0035 (10)
O10A	0.0492 (16)	0.0561 (18)	0.082 (2)	−0.0274 (13)	0.0199 (15)	0.0083 (15)
C1A	0.0186 (12)	0.0211 (12)	0.0144 (11)	−0.0026 (9)	0.0022 (9)	0.0028 (9)
C2A	0.0245 (13)	0.0222 (13)	0.0217 (13)	−0.0034 (10)	0.0001 (10)	0.0021 (10)
C3A	0.0337 (15)	0.0223 (14)	0.0295 (15)	−0.0058 (11)	0.0064 (12)	0.0046 (11)
C4A	0.0279 (15)	0.0358 (17)	0.0261 (15)	−0.0070 (12)	0.0054 (12)	0.0109 (12)
C5A	0.0242 (13)	0.0372 (16)	0.0163 (12)	−0.0062 (11)	0.0049 (10)	0.0019 (11)
C6A	0.0193 (12)	0.0236 (13)	0.0164 (12)	−0.0054 (10)	0.0026 (9)	−0.0013 (10)
C7A	0.0212 (12)	0.0167 (11)	0.0146 (11)	−0.0014 (9)	0.0042 (9)	−0.0023 (9)
C8A	0.0196 (12)	0.0216 (12)	0.0184 (12)	0.0006 (10)	0.0019 (10)	−0.0014 (10)
C9A	0.0223 (13)	0.0299 (15)	0.0225 (14)	0.0002 (11)	−0.0039 (10)	−0.0004 (11)
C10A	0.0398 (17)	0.0275 (15)	0.0157 (13)	−0.0007 (12)	−0.0049 (11)	−0.0009 (11)
C11A	0.0411 (17)	0.0258 (14)	0.0163 (13)	−0.0008 (12)	0.0085 (12)	−0.0017 (10)
C12A	0.0301 (14)	0.0233 (14)	0.0170 (12)	−0.0017 (11)	0.0074 (10)	−0.0014 (10)
C13A	0.0177 (12)	0.0192 (12)	0.0225 (13)	0.0003 (9)	0.0014 (10)	−0.0020 (10)
C14A	0.0191 (11)	0.0204 (12)	0.0144 (11)	0.0028 (9)	0.0028 (9)	0.0001 (9)
C15A	0.0211 (12)	0.0255 (14)	0.0189 (12)	0.0032 (10)	0.0038 (10)	0.0022 (10)
C16A	0.0248 (14)	0.0318 (15)	0.0202 (13)	0.0042 (11)	−0.0002 (10)	−0.0010 (11)
C17A	0.0393 (17)	0.0338 (16)	0.0166 (13)	0.0105 (13)	0.0040 (12)	0.0036 (11)
C18A	0.0392 (17)	0.0330 (16)	0.0238 (14)	0.0058 (13)	0.0163 (13)	0.0064 (12)
C19A	0.0238 (13)	0.0266 (14)	0.0202 (13)	0.0022 (10)	0.0075 (10)	0.0032 (10)
C20A	0.0205 (12)	0.0319 (15)	0.0190 (13)	0.0038 (10)	0.0055 (10)	−0.0075 (11)
C21A	0.0435 (19)	0.0370 (18)	0.0323 (17)	0.0137 (14)	0.0145 (14)	−0.0036 (13)
C22A	0.059 (2)	0.046 (2)	0.049 (2)	0.0193 (18)	0.0177 (19)	−0.0128 (17)
C23A	0.0389 (19)	0.063 (2)	0.0369 (19)	0.0066 (16)	0.0172 (15)	−0.0217 (17)
C24A	0.0305 (16)	0.059 (2)	0.0217 (15)	−0.0043 (14)	0.0117 (12)	−0.0141 (14)
C25A	0.0169 (12)	0.0422 (17)	0.0185 (13)	−0.0017 (11)	0.0049 (10)	−0.0059 (11)
C26A	0.0384 (17)	0.0213 (14)	0.0291 (15)	−0.0058 (12)	0.0066 (13)	−0.0044 (11)
C27A	0.0192 (13)	0.0432 (18)	0.0289 (15)	0.0019 (12)	0.0047 (11)	−0.0063 (13)
C28A	0.0187 (13)	0.0449 (18)	0.0266 (15)	0.0008 (12)	0.0035 (11)	0.0111 (13)
C29A	0.123 (4)	0.034 (2)	0.048 (2)	0.036 (2)	0.032 (2)	0.0086 (17)
C30A	0.0247 (13)	0.0215 (13)	0.0209 (13)	0.0000 (10)	0.0093 (10)	−0.0010 (10)
C31A	0.0203 (12)	0.0208 (13)	0.0245 (13)	−0.0056 (10)	0.0083 (10)	−0.0038 (10)
C32A	0.0261 (13)	0.0257 (14)	0.0163 (12)	0.0010 (10)	0.0069 (10)	−0.0020 (10)
C33A	0.0186 (12)	0.0238 (13)	0.0231 (13)	0.0005 (10)	0.0072 (10)	0.0030 (10)
C34A	0.0229 (13)	0.0213 (13)	0.0229 (13)	−0.0040 (10)	0.0084 (10)	−0.0003 (10)
C35A	0.0272 (14)	0.0311 (15)	0.0172 (13)	−0.0017 (11)	0.0042 (11)	−0.0001 (11)
C36A	0.0451 (18)	0.0233 (15)	0.0306 (16)	−0.0041 (13)	0.0139 (14)	0.0027 (12)
C37A	0.0267 (14)	0.0233 (14)	0.0300 (15)	−0.0001 (11)	0.0074 (12)	−0.0021 (11)
C38A	0.0369 (16)	0.0233 (14)	0.0245 (14)	−0.0017 (12)	0.0112 (12)	0.0000 (11)
C39A	0.0377 (18)	0.0317 (17)	0.0440 (19)	−0.0095 (13)	0.0120 (15)	0.0040 (14)
Ru1B	0.01691 (9)	0.01596 (9)	0.01461 (9)	−0.00020 (7)	0.00479 (7)	−0.00098 (7)
Ru2B	0.01516 (9)	0.01599 (9)	0.01511 (9)	0.00012 (7)	0.00360 (7)	0.00120 (7)
Ru3B	0.02252 (10)	0.01495 (10)	0.02291 (10)	−0.00039 (8)	0.00702 (8)	0.00141 (8)
P1B	0.0145 (3)	0.0167 (3)	0.0140 (3)	−0.0004 (2)	0.0035 (2)	−0.0006 (2)
P2B	0.0142 (3)	0.0167 (3)	0.0160 (3)	0.0008 (2)	0.0029 (2)	0.0012 (2)
O1B	0.0390 (12)	0.0398 (13)	0.0188 (10)	0.0066 (10)	0.0046 (9)	0.0031 (9)
O2B	0.0253 (10)	0.0347 (11)	0.0209 (10)	−0.0059 (8)	0.0034 (8)	0.0001 (8)
O3B	0.0278 (11)	0.0435 (13)	0.0329 (11)	0.0073 (9)	0.0118 (9)	−0.0070 (10)
O4B	0.0276 (10)	0.0290 (11)	0.0191 (9)	0.0013 (8)	0.0028 (8)	−0.0003 (8)
O5B	0.0245 (10)	0.0255 (10)	0.0221 (10)	0.0000 (8)	0.0001 (8)	−0.0010 (8)
O6B	0.0268 (11)	0.0484 (14)	0.0329 (12)	−0.0059 (9)	0.0148 (9)	0.0011 (10)
O7B	0.088 (2)	0.0315 (13)	0.0271 (12)	−0.0007 (12)	0.0163 (12)	0.0076 (10)
O8B	0.0356 (12)	0.0347 (12)	0.0275 (11)	0.0008 (9)	0.0058 (9)	−0.0039 (9)
O9B	0.0365 (13)	0.0320 (12)	0.0586 (15)	0.0115 (10)	0.0156 (11)	0.0031 (11)
O10B	0.0368 (14)	0.0525 (16)	0.0769 (19)	−0.0138 (12)	0.0176 (13)	0.0188 (14)
C1B	0.0213 (12)	0.0166 (11)	0.0154 (11)	−0.0029 (9)	0.0049 (9)	−0.0011 (9)
C2B	0.0185 (12)	0.0221 (13)	0.0193 (12)	−0.0027 (9)	0.0036 (10)	0.0010 (10)
C3B	0.0230 (13)	0.0291 (14)	0.0189 (13)	−0.0050 (11)	−0.0001 (10)	0.0030 (11)
C4B	0.0442 (18)	0.0309 (16)	0.0175 (13)	−0.0134 (13)	0.0069 (12)	−0.0043 (11)
C5B	0.0384 (16)	0.0294 (15)	0.0216 (14)	−0.0102 (12)	0.0156 (12)	−0.0082 (11)
C6B	0.0242 (13)	0.0217 (13)	0.0240 (13)	−0.0030 (10)	0.0106 (11)	−0.0037 (10)
C7B	0.0173 (11)	0.0194 (12)	0.0155 (11)	0.0005 (9)	0.0037 (9)	0.0024 (9)
C8B	0.0216 (12)	0.0197 (12)	0.0216 (13)	0.0008 (10)	0.0050 (10)	0.0011 (10)
C9B	0.0322 (15)	0.0224 (14)	0.0304 (15)	−0.0030 (11)	0.0134 (12)	0.0021 (11)
C10B	0.0335 (15)	0.0291 (15)	0.0263 (14)	0.0029 (12)	0.0135 (12)	0.0097 (12)
C11B	0.0230 (13)	0.0313 (15)	0.0185 (13)	0.0029 (11)	0.0061 (10)	0.0020 (11)
C12B	0.0169 (11)	0.0226 (12)	0.0184 (12)	−0.0005 (9)	0.0038 (9)	0.0011 (10)
C13B	0.0163 (11)	0.0178 (12)	0.0252 (13)	0.0005 (9)	0.0020 (10)	0.0033 (10)
C14B	0.0188 (12)	0.0202 (12)	0.0187 (12)	0.0015 (9)	0.0008 (9)	−0.0024 (10)
C15B	0.0251 (13)	0.0210 (13)	0.0259 (14)	0.0026 (10)	0.0024 (11)	−0.0018 (11)
C16B	0.0465 (19)	0.0236 (15)	0.0312 (16)	0.0081 (13)	0.0067 (14)	−0.0057 (12)
C17B	0.059 (2)	0.0371 (19)	0.0301 (17)	0.0154 (16)	0.0111 (15)	−0.0125 (14)
C18B	0.0442 (18)	0.0375 (18)	0.0184 (14)	0.0064 (14)	0.0066 (13)	−0.0039 (12)
C19B	0.0256 (14)	0.0267 (14)	0.0194 (13)	0.0032 (11)	0.0051 (10)	−0.0010 (10)
C20B	0.0200 (12)	0.0163 (11)	0.0161 (11)	0.0011 (9)	0.0046 (9)	0.0014 (9)
C21B	0.0211 (12)	0.0178 (12)	0.0196 (12)	0.0010 (9)	0.0035 (10)	0.0010 (10)
C22B	0.0229 (13)	0.0224 (13)	0.0227 (13)	0.0021 (10)	−0.0024 (10)	−0.0002 (10)
C23B	0.0380 (17)	0.0267 (15)	0.0188 (13)	0.0042 (12)	0.0007 (12)	0.0003 (11)
C24B	0.0443 (18)	0.0322 (16)	0.0167 (13)	0.0041 (13)	0.0127 (12)	0.0018 (11)
C25B	0.0256 (13)	0.0245 (14)	0.0237 (13)	0.0035 (10)	0.0088 (11)	0.0018 (11)
C26B	0.0192 (13)	0.0375 (16)	0.0233 (14)	0.0043 (11)	0.0017 (11)	−0.0020 (12)
C27B	0.0394 (16)	0.0201 (13)	0.0252 (14)	−0.0091 (11)	0.0105 (12)	−0.0038 (11)
C28B	0.0269 (14)	0.0195 (13)	0.0397 (17)	0.0025 (11)	0.0115 (12)	0.0036 (12)
C29B	0.0170 (12)	0.0331 (16)	0.0260 (14)	0.0019 (11)	0.0043 (10)	0.0028 (11)
C30B	0.0267 (13)	0.0199 (13)	0.0229 (13)	0.0008 (10)	0.0104 (11)	−0.0024 (10)
C31B	0.0194 (12)	0.0192 (12)	0.0217 (13)	−0.0048 (9)	0.0063 (10)	−0.0029 (10)
C32B	0.0245 (13)	0.0252 (14)	0.0177 (12)	−0.0019 (10)	0.0058 (10)	−0.0035 (10)
C33B	0.0196 (12)	0.0193 (12)	0.0233 (13)	0.0015 (9)	0.0060 (10)	0.0038 (10)
C34B	0.0175 (11)	0.0167 (12)	0.0220 (13)	−0.0012 (9)	0.0056 (10)	0.0002 (10)
C35B	0.0213 (12)	0.0255 (13)	0.0199 (13)	−0.0013 (10)	0.0027 (10)	0.0012 (10)
C36B	0.0477 (19)	0.0186 (14)	0.0315 (16)	−0.0011 (12)	0.0124 (14)	0.0040 (12)
C37B	0.0250 (13)	0.0214 (13)	0.0284 (14)	0.0014 (10)	0.0089 (11)	0.0001 (11)
C38B	0.0308 (15)	0.0212 (14)	0.0303 (15)	−0.0018 (11)	0.0090 (12)	0.0010 (11)
C39B	0.0264 (15)	0.0307 (16)	0.0398 (17)	−0.0023 (12)	0.0090 (13)	0.0072 (13)

### Geometric parameters (Å, °)

**Table:**

Ru1A—C32A	1.902 (3)	Ru1B—P1B	2.3450 (7)
Ru1A—C30A	1.933 (3)	Ru1B—Ru2B	2.8429 (3)
Ru1A—C31A	1.934 (3)	Ru1B—Ru3B	2.8554 (3)
Ru1A—P1A	2.3451 (7)	Ru2B—C35B	1.895 (3)
Ru1A—Ru2A	2.8350 (3)	Ru2B—C34B	1.931 (3)
Ru1A—Ru3A	2.8420 (3)	Ru2B—C33B	1.931 (3)
Ru2A—C35A	1.892 (3)	Ru2B—P2B	2.3459 (7)
Ru2A—C33A	1.927 (3)	Ru2B—Ru3B	2.8442 (3)
Ru2A—C34A	1.935 (3)	Ru3B—C38B	1.909 (3)
Ru2A—P2A	2.3422 (7)	Ru3B—C39B	1.923 (3)
Ru2A—Ru3A	2.8280 (3)	Ru3B—C36B	1.941 (3)
Ru3A—C38A	1.906 (3)	Ru3B—C37B	1.953 (3)
Ru3A—C39A	1.918 (3)	P1B—C1B	1.831 (2)
Ru3A—C36A	1.940 (3)	P1B—C13B	1.850 (2)
Ru3A—C37A	1.954 (3)	P1B—C7B	1.855 (2)
P1A—C7A	1.842 (2)	P2B—C14B	1.840 (3)
P1A—C1A	1.843 (2)	P2B—C20B	1.842 (2)
P1A—C13A	1.869 (3)	P2B—C13B	1.865 (3)
P2A—C14A	1.832 (2)	O1B—C30B	1.141 (3)
P2A—C13A	1.843 (3)	O2B—C31B	1.144 (3)
P2A—C20A	1.846 (3)	O3B—C32B	1.146 (3)
O1A—C30A	1.141 (3)	O4B—C33B	1.146 (3)
O2A—C31A	1.145 (3)	O5B—C34B	1.142 (3)
O3A—C32A	1.139 (3)	O6B—C35B	1.150 (3)
O4A—C33A	1.146 (3)	O7B—C36B	1.132 (3)
O5A—C34A	1.143 (3)	O8B—C37B	1.131 (3)
O6A—C35A	1.155 (3)	O9B—C38B	1.145 (3)
O7A—C36A	1.134 (3)	O10B—C39B	1.123 (3)
O8A—C37A	1.131 (3)	C1B—C6B	1.398 (3)
O9A—C38A	1.147 (3)	C1B—C2B	1.404 (3)
O10A—C39A	1.131 (4)	C2B—C3B	1.400 (4)
C1A—C6A	1.397 (3)	C2B—C26B	1.507 (4)
C1A—C2A	1.410 (4)	C3B—C4B	1.381 (4)
C2A—C3A	1.395 (4)	C3B—H3BA	0.9300
C2A—C26A	1.513 (4)	C4B—C5B	1.384 (4)
C3A—C4A	1.381 (4)	C4B—H4BA	0.9300
C3A—H3AA	0.9300	C5B—C6B	1.387 (4)
C4A—C5A	1.374 (4)	C5B—H5BA	0.9300
C4A—H4AA	0.9300	C6B—H6BA	0.9300
C5A—C6A	1.386 (3)	C7B—C12B	1.396 (3)
C5A—H5AA	0.9300	C7B—C8B	1.417 (3)
C6A—H6AA	0.9300	C8B—C9B	1.383 (4)
C7A—C12A	1.401 (3)	C8B—C27B	1.513 (4)
C7A—C8A	1.406 (3)	C9B—C10B	1.396 (4)
C8A—C9A	1.394 (3)	C9B—H9BA	0.9300
C8A—C27A	1.510 (4)	C10B—C11B	1.382 (4)
C9A—C10A	1.373 (4)	C10B—H10B	0.9300
C9A—H9AA	0.9300	C11B—C12B	1.388 (3)
C10A—C11A	1.394 (4)	C11B—H11B	0.9300
C10A—H10A	0.9300	C12B—H12B	0.9300
C11A—C12A	1.389 (4)	C13B—H13C	0.9700
C11A—H11A	0.9300	C13B—H13D	0.9700
C12A—H12A	0.9300	C14B—C19B	1.400 (4)
C13A—H13A	0.9700	C14B—C15B	1.412 (4)
C13A—H13B	0.9700	C15B—C16B	1.391 (4)
C14A—C19A	1.399 (3)	C15B—C28B	1.514 (4)
C14A—C15A	1.407 (3)	C16B—C17B	1.370 (4)
C15A—C16A	1.395 (4)	C16B—H16B	0.9300
C15A—C28A	1.507 (4)	C17B—C18B	1.374 (4)
C16A—C17A	1.372 (4)	C17B—H17B	0.9300
C16A—H16A	0.9300	C18B—C19B	1.382 (4)
C17A—C18A	1.390 (4)	C18B—H18B	0.9300
C17A—H17A	0.9300	C19B—H19B	0.9300
C18A—C19A	1.389 (4)	C20B—C21B	1.401 (3)
C18A—H18A	0.9300	C20B—C25B	1.407 (3)
C19A—H19A	0.9300	C21B—C22B	1.398 (3)
C20A—C25A	1.394 (4)	C21B—C29B	1.517 (4)
C20A—C21A	1.404 (4)	C22B—C23B	1.376 (4)
C21A—C22A	1.394 (4)	C22B—H22B	0.9300
C21A—C29A	1.513 (5)	C23B—C24B	1.389 (4)
C22A—C23A	1.391 (5)	C23B—H23B	0.9300
C22A—H22A	0.9300	C24B—C25B	1.386 (4)
C23A—C24A	1.368 (5)	C24B—H24B	0.9300
C23A—H23A	0.9300	C25B—H25B	0.9300
C24A—C25A	1.395 (4)	C26B—H26D	0.9600
C24A—H24A	0.9300	C26B—H26E	0.9600
C25A—H25A	0.9300	C26B—H26F	0.9600
C26A—H26A	0.9600	C27B—H27D	0.9600
C26A—H26B	0.9600	C27B—H27E	0.9600
C26A—H26C	0.9600	C27B—H27F	0.9600
C27A—H27A	0.9600	C28B—H28D	0.9600
C27A—H27B	0.9600	C28B—H28E	0.9600
C27A—H27C	0.9600	C28B—H28F	0.9600
C28A—H28A	0.9600	C29B—H29D	0.9600
C28A—H28B	0.9600	C29B—H29E	0.9600
C28A—H28C	0.9600	C29B—H29F	0.9600
C29A—H29A	0.9600	O11—C40	1.452 (9)
C29A—H29B	0.9600	O11—H11O	0.8200
C29A—H29C	0.9600	C40—H40A	0.9600
Ru1B—C32B	1.894 (3)	C40—H40B	0.9600
Ru1B—C30B	1.931 (3)	C40—H40C	0.9600
Ru1B—C31B	1.936 (3)		
			
C32A—Ru1A—C30A	88.63 (11)	C30B—Ru1B—P1B	91.44 (8)
C32A—Ru1A—C31A	91.96 (11)	C31B—Ru1B—P1B	95.74 (8)
C30A—Ru1A—C31A	173.81 (11)	C32B—Ru1B—Ru2B	165.78 (8)
C32A—Ru1A—P1A	103.18 (8)	C30B—Ru1B—Ru2B	95.00 (8)
C30A—Ru1A—P1A	91.17 (8)	C31B—Ru1B—Ru2B	83.35 (7)
C31A—Ru1A—P1A	94.69 (8)	P1B—Ru1B—Ru2B	91.367 (17)
C32A—Ru1A—Ru2A	162.77 (8)	C32B—Ru1B—Ru3B	107.27 (8)
C30A—Ru1A—Ru2A	96.43 (8)	C30B—Ru1B—Ru3B	82.15 (8)
C31A—Ru1A—Ru2A	81.29 (7)	C31B—Ru1B—Ru3B	90.87 (7)
P1A—Ru1A—Ru2A	93.205 (17)	P1B—Ru1B—Ru3B	149.568 (17)
C32A—Ru1A—Ru3A	104.63 (8)	Ru2B—Ru1B—Ru3B	59.886 (7)
C30A—Ru1A—Ru3A	84.31 (8)	C35B—Ru2B—C34B	89.26 (11)
C31A—Ru1A—Ru3A	89.57 (7)	C35B—Ru2B—C33B	91.97 (11)
P1A—Ru1A—Ru3A	151.682 (18)	C34B—Ru2B—C33B	175.99 (11)
Ru2A—Ru1A—Ru3A	59.755 (7)	C35B—Ru2B—P2B	105.74 (8)
C35A—Ru2A—C33A	92.38 (11)	C34B—Ru2B—P2B	89.41 (7)
C35A—Ru2A—C34A	89.15 (11)	C33B—Ru2B—P2B	93.92 (8)
C33A—Ru2A—C34A	176.84 (11)	C35B—Ru2B—Ru1B	160.45 (8)
C35A—Ru2A—P2A	106.36 (9)	C34B—Ru2B—Ru1B	93.99 (7)
C33A—Ru2A—P2A	91.90 (8)	C33B—Ru2B—Ru1B	83.58 (7)
C34A—Ru2A—P2A	90.32 (8)	P2B—Ru2B—Ru1B	93.579 (17)
C35A—Ru2A—Ru3A	101.80 (9)	C35B—Ru2B—Ru3B	101.25 (8)
C33A—Ru2A—Ru3A	93.94 (8)	C34B—Ru2B—Ru3B	82.08 (7)
C34A—Ru2A—Ru3A	83.04 (8)	C33B—Ru2B—Ru3B	93.94 (8)
P2A—Ru2A—Ru3A	150.948 (18)	P2B—Ru2B—Ru3B	151.558 (18)
C35A—Ru2A—Ru1A	160.20 (9)	Ru1B—Ru2B—Ru3B	60.275 (7)
C33A—Ru2A—Ru1A	81.39 (7)	C38B—Ru3B—C39B	104.77 (13)
C34A—Ru2A—Ru1A	96.26 (8)	C38B—Ru3B—C36B	93.83 (13)
P2A—Ru2A—Ru1A	92.682 (17)	C39B—Ru3B—C36B	88.26 (13)
Ru3A—Ru2A—Ru1A	60.246 (7)	C38B—Ru3B—C37B	91.40 (12)
C38A—Ru3A—C39A	106.69 (14)	C39B—Ru3B—C37B	89.77 (12)
C38A—Ru3A—C36A	92.55 (13)	C36B—Ru3B—C37B	174.73 (12)
C39A—Ru3A—C36A	89.22 (14)	C38B—Ru3B—Ru2B	152.18 (9)
C38A—Ru3A—C37A	92.02 (12)	C39B—Ru3B—Ru2B	102.29 (9)
C39A—Ru3A—C37A	88.92 (13)	C36B—Ru3B—Ru2B	80.57 (9)
C36A—Ru3A—C37A	175.40 (13)	C37B—Ru3B—Ru2B	95.08 (8)
C38A—Ru3A—Ru2A	151.28 (9)	C38B—Ru3B—Ru1B	93.62 (8)
C39A—Ru3A—Ru2A	101.28 (10)	C39B—Ru3B—Ru1B	161.40 (9)
C36A—Ru3A—Ru2A	81.29 (9)	C36B—Ru3B—Ru1B	93.28 (9)
C37A—Ru3A—Ru2A	94.94 (8)	C37B—Ru3B—Ru1B	87.04 (8)
C38A—Ru3A—Ru1A	92.61 (9)	Ru2B—Ru3B—Ru1B	59.839 (8)
C39A—Ru3A—Ru1A	160.47 (10)	C1B—P1B—C13B	104.00 (12)
C36A—Ru3A—Ru1A	92.96 (9)	C1B—P1B—C7B	107.70 (11)
C37A—Ru3A—Ru1A	87.40 (8)	C13B—P1B—C7B	96.33 (11)
Ru2A—Ru3A—Ru1A	59.999 (8)	C1B—P1B—Ru1B	112.59 (8)
C7A—P1A—C1A	106.67 (11)	C13B—P1B—Ru1B	114.74 (8)
C7A—P1A—C13A	102.74 (11)	C7B—P1B—Ru1B	119.41 (8)
C1A—P1A—C13A	99.85 (12)	C14B—P2B—C20B	105.50 (11)
C7A—P1A—Ru1A	112.52 (8)	C14B—P2B—C13B	100.68 (12)
C1A—P1A—Ru1A	118.21 (8)	C20B—P2B—C13B	103.39 (11)
C13A—P1A—Ru1A	115.05 (8)	C14B—P2B—Ru2B	119.59 (9)
C14A—P2A—C13A	104.04 (12)	C20B—P2B—Ru2B	111.42 (8)
C14A—P2A—C20A	107.04 (12)	C13B—P2B—Ru2B	114.50 (8)
C13A—P2A—C20A	99.22 (12)	C6B—C1B—C2B	119.4 (2)
C14A—P2A—Ru2A	113.14 (8)	C6B—C1B—P1B	119.59 (19)
C13A—P2A—Ru2A	114.00 (8)	C2B—C1B—P1B	120.94 (19)
C20A—P2A—Ru2A	117.72 (10)	C3B—C2B—C1B	118.2 (2)
C6A—C1A—C2A	119.0 (2)	C3B—C2B—C26B	118.7 (2)
C6A—C1A—P1A	117.20 (19)	C1B—C2B—C26B	123.1 (2)
C2A—C1A—P1A	123.71 (19)	C4B—C3B—C2B	121.9 (3)
C3A—C2A—C1A	117.7 (3)	C4B—C3B—H3BA	119.1
C3A—C2A—C26A	117.7 (2)	C2B—C3B—H3BA	119.1
C1A—C2A—C26A	124.6 (2)	C3B—C4B—C5B	119.9 (3)
C4A—C3A—C2A	122.4 (3)	C3B—C4B—H4BA	120.1
C4A—C3A—H3AA	118.8	C5B—C4B—H4BA	120.1
C2A—C3A—H3AA	118.8	C4B—C5B—C6B	119.3 (3)
C5A—C4A—C3A	120.0 (3)	C4B—C5B—H5BA	120.3
C5A—C4A—H4AA	120.0	C6B—C5B—H5BA	120.3
C3A—C4A—H4AA	120.0	C5B—C6B—C1B	121.4 (3)
C4A—C5A—C6A	118.9 (3)	C5B—C6B—H6BA	119.3
C4A—C5A—H5AA	120.6	C1B—C6B—H6BA	119.3
C6A—C5A—H5AA	120.6	C12B—C7B—C8B	119.2 (2)
C5A—C6A—C1A	122.1 (2)	C12B—C7B—P1B	114.88 (18)
C5A—C6A—H6AA	119.0	C8B—C7B—P1B	125.31 (19)
C1A—C6A—H6AA	119.0	C9B—C8B—C7B	117.6 (2)
C12A—C7A—C8A	119.1 (2)	C9B—C8B—C27B	117.5 (2)
C12A—C7A—P1A	119.11 (19)	C7B—C8B—C27B	124.9 (2)
C8A—C7A—P1A	121.65 (18)	C8B—C9B—C10B	123.0 (3)
C9A—C8A—C7A	118.0 (2)	C8B—C9B—H9BA	118.5
C9A—C8A—C27A	118.3 (2)	C10B—C9B—H9BA	118.5
C7A—C8A—C27A	123.7 (2)	C11B—C10B—C9B	119.1 (3)
C10A—C9A—C8A	122.8 (3)	C11B—C10B—H10B	120.5
C10A—C9A—H9AA	118.6	C9B—C10B—H10B	120.5
C8A—C9A—H9AA	118.6	C10B—C11B—C12B	119.2 (3)
C9A—C10A—C11A	119.4 (2)	C10B—C11B—H11B	120.4
C9A—C10A—H10A	120.3	C12B—C11B—H11B	120.4
C11A—C10A—H10A	120.3	C11B—C12B—C7B	121.9 (2)
C12A—C11A—C10A	119.1 (3)	C11B—C12B—H12B	119.0
C12A—C11A—H11A	120.5	C7B—C12B—H12B	119.0
C10A—C11A—H11A	120.5	P1B—C13B—P2B	117.59 (14)
C11A—C12A—C7A	121.6 (3)	P1B—C13B—H13C	107.9
C11A—C12A—H12A	119.2	P2B—C13B—H13C	107.9
C7A—C12A—H12A	119.2	P1B—C13B—H13D	107.9
P2A—C13A—P1A	118.00 (14)	P2B—C13B—H13D	107.9
P2A—C13A—H13A	107.8	H13C—C13B—H13D	107.2
P1A—C13A—H13A	107.8	C19B—C14B—C15B	119.1 (2)
P2A—C13A—H13B	107.8	C19B—C14B—P2B	117.6 (2)
P1A—C13A—H13B	107.8	C15B—C14B—P2B	123.3 (2)
H13A—C13A—H13B	107.1	C16B—C15B—C14B	117.6 (3)
C19A—C14A—C15A	119.4 (2)	C16B—C15B—C28B	118.4 (3)
C19A—C14A—P2A	119.82 (19)	C14B—C15B—C28B	124.1 (2)
C15A—C14A—P2A	120.64 (19)	C17B—C16B—C15B	122.6 (3)
C16A—C15A—C14A	118.1 (2)	C17B—C16B—H16B	118.7
C16A—C15A—C28A	118.8 (2)	C15B—C16B—H16B	118.7
C14A—C15A—C28A	123.0 (2)	C16B—C17B—C18B	119.9 (3)
C17A—C16A—C15A	122.2 (3)	C16B—C17B—H17B	120.1
C17A—C16A—H16A	118.9	C18B—C17B—H17B	120.1
C15A—C16A—H16A	118.9	C17B—C18B—C19B	119.4 (3)
C16A—C17A—C18A	119.8 (3)	C17B—C18B—H18B	120.3
C16A—C17A—H17A	120.1	C19B—C18B—H18B	120.3
C18A—C17A—H17A	120.1	C18B—C19B—C14B	121.4 (3)
C19A—C18A—C17A	119.4 (3)	C18B—C19B—H19B	119.3
C19A—C18A—H18A	120.3	C14B—C19B—H19B	119.3
C17A—C18A—H18A	120.3	C21B—C20B—C25B	118.8 (2)
C18A—C19A—C14A	121.0 (3)	C21B—C20B—P2B	120.95 (18)
C18A—C19A—H19A	119.5	C25B—C20B—P2B	120.01 (19)
C14A—C19A—H19A	119.5	C22B—C21B—C20B	118.6 (2)
C25A—C20A—C21A	118.7 (3)	C22B—C21B—C29B	117.7 (2)
C25A—C20A—P2A	115.9 (2)	C20B—C21B—C29B	123.7 (2)
C21A—C20A—P2A	125.3 (2)	C23B—C22B—C21B	122.3 (3)
C22A—C21A—C20A	118.3 (3)	C23B—C22B—H22B	118.8
C22A—C21A—C29A	118.0 (3)	C21B—C22B—H22B	118.8
C20A—C21A—C29A	123.7 (3)	C22B—C23B—C24B	119.2 (3)
C23A—C22A—C21A	122.1 (3)	C22B—C23B—H23B	120.4
C23A—C22A—H22A	118.9	C24B—C23B—H23B	120.4
C21A—C22A—H22A	118.9	C25B—C24B—C23B	119.7 (3)
C24A—C23A—C22A	119.7 (3)	C25B—C24B—H24B	120.1
C24A—C23A—H23A	120.2	C23B—C24B—H24B	120.1
C22A—C23A—H23A	120.2	C24B—C25B—C20B	121.3 (3)
C23A—C24A—C25A	119.1 (3)	C24B—C25B—H25B	119.4
C23A—C24A—H24A	120.5	C20B—C25B—H25B	119.4
C25A—C24A—H24A	120.5	C2B—C26B—H26D	109.5
C20A—C25A—C24A	122.1 (3)	C2B—C26B—H26E	109.5
C20A—C25A—H25A	118.9	H26D—C26B—H26E	109.5
C24A—C25A—H25A	118.9	C2B—C26B—H26F	109.5
C2A—C26A—H26A	109.5	H26D—C26B—H26F	109.5
C2A—C26A—H26B	109.5	H26E—C26B—H26F	109.5
H26A—C26A—H26B	109.5	C8B—C27B—H27D	109.5
C2A—C26A—H26C	109.5	C8B—C27B—H27E	109.5
H26A—C26A—H26C	109.5	H27D—C27B—H27E	109.5
H26B—C26A—H26C	109.5	C8B—C27B—H27F	109.5
C8A—C27A—H27A	109.5	H27D—C27B—H27F	109.5
C8A—C27A—H27B	109.5	H27E—C27B—H27F	109.5
H27A—C27A—H27B	109.5	C15B—C28B—H28D	109.5
C8A—C27A—H27C	109.5	C15B—C28B—H28E	109.5
H27A—C27A—H27C	109.5	H28D—C28B—H28E	109.5
H27B—C27A—H27C	109.5	C15B—C28B—H28F	109.5
C15A—C28A—H28A	109.5	H28D—C28B—H28F	109.5
C15A—C28A—H28B	109.5	H28E—C28B—H28F	109.5
H28A—C28A—H28B	109.5	C21B—C29B—H29D	109.5
C15A—C28A—H28C	109.5	C21B—C29B—H29E	109.5
H28A—C28A—H28C	109.5	H29D—C29B—H29E	109.5
H28B—C28A—H28C	109.5	C21B—C29B—H29F	109.5
C21A—C29A—H29A	109.5	H29D—C29B—H29F	109.5
C21A—C29A—H29B	109.5	H29E—C29B—H29F	109.5
H29A—C29A—H29B	109.5	O1B—C30B—Ru1B	172.9 (2)
C21A—C29A—H29C	109.5	O2B—C31B—Ru1B	173.6 (2)
H29A—C29A—H29C	109.5	O3B—C32B—Ru1B	178.7 (2)
H29B—C29A—H29C	109.5	O4B—C33B—Ru2B	173.8 (2)
O1A—C30A—Ru1A	173.6 (2)	O5B—C34B—Ru2B	173.5 (2)
O2A—C31A—Ru1A	173.0 (2)	O6B—C35B—Ru2B	178.1 (3)
O3A—C32A—Ru1A	179.4 (3)	O7B—C36B—Ru3B	171.9 (3)
O4A—C33A—Ru2A	173.2 (2)	O8B—C37B—Ru3B	172.4 (2)
O5A—C34A—Ru2A	172.5 (2)	O9B—C38B—Ru3B	178.4 (3)
O6A—C35A—Ru2A	178.7 (3)	O10B—C39B—Ru3B	178.6 (3)
O7A—C36A—Ru3A	172.2 (3)	C40—O11—H11O	109.5
O8A—C37A—Ru3A	172.4 (3)	O11—C40—H40A	109.5
O9A—C38A—Ru3A	178.2 (3)	O11—C40—H40B	109.5
O10A—C39A—Ru3A	179.1 (3)	H40A—C40—H40B	109.5
C32B—Ru1B—C30B	88.74 (11)	O11—C40—H40C	109.5
C32B—Ru1B—C31B	91.19 (11)	H40A—C40—H40C	109.5
C30B—Ru1B—C31B	172.66 (11)	H40B—C40—H40C	109.5
C32B—Ru1B—P1B	102.27 (8)		
			
C32A—Ru1A—Ru2A—C35A	53.6 (4)	C32B—Ru1B—Ru2B—C35B	−47.5 (4)
C30A—Ru1A—Ru2A—C35A	−52.7 (3)	C30B—Ru1B—Ru2B—C35B	57.3 (3)
C31A—Ru1A—Ru2A—C35A	121.5 (3)	C31B—Ru1B—Ru2B—C35B	−115.5 (2)
P1A—Ru1A—Ru2A—C35A	−144.3 (2)	P1B—Ru1B—Ru2B—C35B	148.9 (2)
Ru3A—Ru1A—Ru2A—C35A	26.8 (2)	Ru3B—Ru1B—Ru2B—C35B	−20.6 (2)
C32A—Ru1A—Ru2A—C33A	126.4 (3)	C32B—Ru1B—Ru2B—C34B	51.6 (3)
C30A—Ru1A—Ru2A—C33A	20.02 (11)	C30B—Ru1B—Ru2B—C34B	156.44 (11)
C31A—Ru1A—Ru2A—C33A	−165.78 (11)	C31B—Ru1B—Ru2B—C34B	−16.36 (10)
P1A—Ru1A—Ru2A—C33A	−71.52 (8)	P1B—Ru1B—Ru2B—C34B	−111.99 (8)
Ru3A—Ru1A—Ru2A—C33A	99.59 (8)	Ru3B—Ru1B—Ru2B—C34B	78.53 (7)
C32A—Ru1A—Ru2A—C34A	−51.5 (3)	C32B—Ru1B—Ru2B—C33B	−125.2 (3)
C30A—Ru1A—Ru2A—C34A	−157.84 (11)	C30B—Ru1B—Ru2B—C33B	−20.35 (11)
C31A—Ru1A—Ru2A—C34A	16.35 (11)	C31B—Ru1B—Ru2B—C33B	166.84 (11)
P1A—Ru1A—Ru2A—C34A	110.61 (8)	P1B—Ru1B—Ru2B—C33B	71.22 (8)
Ru3A—Ru1A—Ru2A—C34A	−78.28 (8)	Ru3B—Ru1B—Ru2B—C33B	−98.27 (8)
C32A—Ru1A—Ru2A—P2A	−142.1 (3)	C32B—Ru1B—Ru2B—P2B	141.3 (3)
C30A—Ru1A—Ru2A—P2A	111.54 (8)	C30B—Ru1B—Ru2B—P2B	−113.90 (8)
C31A—Ru1A—Ru2A—P2A	−74.27 (8)	C31B—Ru1B—Ru2B—P2B	73.29 (8)
P1A—Ru1A—Ru2A—P2A	20.00 (2)	P1B—Ru1B—Ru2B—P2B	−22.33 (2)
Ru3A—Ru1A—Ru2A—P2A	−168.890 (18)	Ru3B—Ru1B—Ru2B—P2B	168.180 (18)
C32A—Ru1A—Ru2A—Ru3A	26.8 (3)	C32B—Ru1B—Ru2B—Ru3B	−26.9 (3)
C30A—Ru1A—Ru2A—Ru3A	−79.57 (8)	C30B—Ru1B—Ru2B—Ru3B	77.92 (8)
C31A—Ru1A—Ru2A—Ru3A	94.62 (8)	C31B—Ru1B—Ru2B—Ru3B	−94.89 (7)
P1A—Ru1A—Ru2A—Ru3A	−171.113 (17)	P1B—Ru1B—Ru2B—Ru3B	169.488 (18)
C35A—Ru2A—Ru3A—C38A	−151.75 (19)	C35B—Ru2B—Ru3B—C38B	153.9 (2)
C33A—Ru2A—Ru3A—C38A	−58.48 (19)	C34B—Ru2B—Ru3B—C38B	−118.4 (2)
C34A—Ru2A—Ru3A—C38A	120.59 (19)	C33B—Ru2B—Ru3B—C38B	61.1 (2)
P2A—Ru2A—Ru3A—C38A	42.61 (18)	P2B—Ru2B—Ru3B—C38B	−44.60 (19)
Ru1A—Ru2A—Ru3A—C38A	19.26 (17)	Ru1B—Ru2B—Ru3B—C38B	−19.18 (18)
C35A—Ru2A—Ru3A—C39A	14.99 (13)	C35B—Ru2B—Ru3B—C39B	−12.43 (12)
C33A—Ru2A—Ru3A—C39A	108.25 (13)	C34B—Ru2B—Ru3B—C39B	75.23 (12)
C34A—Ru2A—Ru3A—C39A	−72.68 (13)	C33B—Ru2B—Ru3B—C39B	−105.23 (12)
P2A—Ru2A—Ru3A—C39A	−150.65 (11)	P2B—Ru2B—Ru3B—C39B	149.04 (10)
Ru1A—Ru2A—Ru3A—C39A	−174.00 (10)	Ru1B—Ru2B—Ru3B—C39B	174.46 (10)
C35A—Ru2A—Ru3A—C36A	−72.46 (12)	C35B—Ru2B—Ru3B—C36B	73.70 (12)
C33A—Ru2A—Ru3A—C36A	20.81 (12)	C34B—Ru2B—Ru3B—C36B	161.35 (12)
C34A—Ru2A—Ru3A—C36A	−160.12 (12)	C33B—Ru2B—Ru3B—C36B	−19.11 (12)
P2A—Ru2A—Ru3A—C36A	121.90 (10)	P2B—Ru2B—Ru3B—C36B	−124.84 (10)
Ru1A—Ru2A—Ru3A—C36A	98.55 (9)	Ru1B—Ru2B—Ru3B—C36B	−99.42 (10)
C35A—Ru2A—Ru3A—C37A	104.87 (12)	C35B—Ru2B—Ru3B—C37B	−103.30 (11)
C33A—Ru2A—Ru3A—C37A	−161.86 (11)	C34B—Ru2B—Ru3B—C37B	−15.64 (11)
C34A—Ru2A—Ru3A—C37A	17.21 (11)	C33B—Ru2B—Ru3B—C37B	163.90 (11)
P2A—Ru2A—Ru3A—C37A	−60.77 (9)	P2B—Ru2B—Ru3B—C37B	58.17 (9)
Ru1A—Ru2A—Ru3A—C37A	−84.12 (8)	Ru1B—Ru2B—Ru3B—C37B	83.59 (8)
C35A—Ru2A—Ru3A—Ru1A	−171.01 (8)	C35B—Ru2B—Ru3B—Ru1B	173.11 (8)
C33A—Ru2A—Ru3A—Ru1A	−77.74 (7)	C34B—Ru2B—Ru3B—Ru1B	−99.23 (7)
C34A—Ru2A—Ru3A—Ru1A	101.33 (7)	C33B—Ru2B—Ru3B—Ru1B	80.31 (7)
P2A—Ru2A—Ru3A—Ru1A	23.35 (4)	P2B—Ru2B—Ru3B—Ru1B	−25.42 (4)
C32A—Ru1A—Ru3A—C38A	17.06 (12)	C32B—Ru1B—Ru3B—C38B	−15.52 (12)
C30A—Ru1A—Ru3A—C38A	−70.02 (11)	C30B—Ru1B—Ru3B—C38B	70.69 (12)
C31A—Ru1A—Ru3A—C38A	108.97 (11)	C31B—Ru1B—Ru3B—C38B	−107.04 (11)
P1A—Ru1A—Ru3A—C38A	−151.89 (9)	P1B—Ru1B—Ru3B—C38B	150.06 (9)
Ru2A—Ru1A—Ru3A—C38A	−170.87 (8)	Ru2B—Ru1B—Ru3B—C38B	171.16 (9)
C32A—Ru1A—Ru3A—C39A	−154.2 (3)	C32B—Ru1B—Ru3B—C39B	156.1 (3)
C30A—Ru1A—Ru3A—C39A	118.7 (3)	C30B—Ru1B—Ru3B—C39B	−117.7 (3)
C31A—Ru1A—Ru3A—C39A	−62.3 (3)	C31B—Ru1B—Ru3B—C39B	64.6 (3)
P1A—Ru1A—Ru3A—C39A	36.8 (3)	P1B—Ru1B—Ru3B—C39B	−38.3 (3)
Ru2A—Ru1A—Ru3A—C39A	17.8 (3)	Ru2B—Ru1B—Ru3B—C39B	−17.2 (3)
C32A—Ru1A—Ru3A—C36A	109.75 (12)	C32B—Ru1B—Ru3B—C36B	−109.58 (12)
C30A—Ru1A—Ru3A—C36A	22.67 (12)	C30B—Ru1B—Ru3B—C36B	−23.36 (12)
C31A—Ru1A—Ru3A—C36A	−158.34 (12)	C31B—Ru1B—Ru3B—C36B	158.91 (12)
P1A—Ru1A—Ru3A—C36A	−59.20 (10)	P1B—Ru1B—Ru3B—C36B	56.00 (10)
Ru2A—Ru1A—Ru3A—C36A	−78.18 (10)	Ru2B—Ru1B—Ru3B—C36B	77.11 (9)
C32A—Ru1A—Ru3A—C37A	−74.84 (11)	C32B—Ru1B—Ru3B—C37B	75.69 (11)
C30A—Ru1A—Ru3A—C37A	−161.92 (11)	C30B—Ru1B—Ru3B—C37B	161.91 (11)
C31A—Ru1A—Ru3A—C37A	17.07 (11)	C31B—Ru1B—Ru3B—C37B	−15.82 (11)
P1A—Ru1A—Ru3A—C37A	116.20 (9)	P1B—Ru1B—Ru3B—C37B	−118.73 (9)
Ru2A—Ru1A—Ru3A—C37A	97.23 (8)	Ru2B—Ru1B—Ru3B—C37B	−97.62 (8)
C32A—Ru1A—Ru3A—Ru2A	−172.07 (8)	C32B—Ru1B—Ru3B—Ru2B	173.32 (8)
C30A—Ru1A—Ru3A—Ru2A	100.85 (8)	C30B—Ru1B—Ru3B—Ru2B	−100.47 (8)
C31A—Ru1A—Ru3A—Ru2A	−80.16 (8)	C31B—Ru1B—Ru3B—Ru2B	81.80 (8)
P1A—Ru1A—Ru3A—Ru2A	18.98 (4)	P1B—Ru1B—Ru3B—Ru2B	−21.10 (3)
C32A—Ru1A—P1A—C7A	−81.13 (12)	C32B—Ru1B—P1B—C1B	93.21 (12)
C30A—Ru1A—P1A—C7A	7.74 (11)	C30B—Ru1B—P1B—C1B	4.18 (12)
C31A—Ru1A—P1A—C7A	−174.25 (11)	C31B—Ru1B—P1B—C1B	−174.31 (11)
Ru2A—Ru1A—P1A—C7A	104.24 (9)	Ru2B—Ru1B—P1B—C1B	−90.86 (9)
Ru3A—Ru1A—P1A—C7A	87.90 (9)	Ru3B—Ru1B—P1B—C1B	−72.70 (10)
C32A—Ru1A—P1A—C1A	43.95 (12)	C32B—Ru1B—P1B—C13B	−148.09 (12)
C30A—Ru1A—P1A—C1A	132.82 (12)	C30B—Ru1B—P1B—C13B	122.88 (12)
C31A—Ru1A—P1A—C1A	−49.17 (12)	C31B—Ru1B—P1B—C13B	−55.61 (12)
Ru2A—Ru1A—P1A—C1A	−130.68 (9)	Ru2B—Ru1B—P1B—C13B	27.84 (10)
Ru3A—Ru1A—P1A—C1A	−147.02 (9)	Ru3B—Ru1B—P1B—C13B	46.00 (11)
C32A—Ru1A—P1A—C13A	161.67 (12)	C32B—Ru1B—P1B—C7B	−34.56 (12)
C30A—Ru1A—P1A—C13A	−109.47 (12)	C30B—Ru1B—P1B—C7B	−123.59 (12)
C31A—Ru1A—P1A—C13A	68.54 (12)	C31B—Ru1B—P1B—C7B	57.91 (12)
Ru2A—Ru1A—P1A—C13A	−12.97 (9)	Ru2B—Ru1B—P1B—C7B	141.37 (9)
Ru3A—Ru1A—P1A—C13A	−29.31 (11)	Ru3B—Ru1B—P1B—C7B	159.52 (9)
C35A—Ru2A—P2A—C14A	−93.15 (12)	C35B—Ru2B—P2B—C14B	−41.26 (12)
C33A—Ru2A—P2A—C14A	173.81 (11)	C34B—Ru2B—P2B—C14B	−130.33 (12)
C34A—Ru2A—P2A—C14A	−3.94 (12)	C33B—Ru2B—P2B—C14B	51.90 (12)
Ru3A—Ru2A—P2A—C14A	72.19 (10)	Ru1B—Ru2B—P2B—C14B	135.70 (9)
Ru1A—Ru2A—P2A—C14A	92.34 (9)	Ru3B—Ru2B—P2B—C14B	157.64 (9)
C35A—Ru2A—P2A—C13A	148.23 (12)	C35B—Ru2B—P2B—C20B	82.31 (12)
C33A—Ru2A—P2A—C13A	55.18 (12)	C34B—Ru2B—P2B—C20B	−6.76 (11)
C34A—Ru2A—P2A—C13A	−122.56 (12)	C33B—Ru2B—P2B—C20B	175.47 (11)
Ru3A—Ru2A—P2A—C13A	−46.43 (10)	Ru1B—Ru2B—P2B—C20B	−100.73 (9)
Ru1A—Ru2A—P2A—C13A	−26.28 (9)	Ru3B—Ru2B—P2B—C20B	−78.79 (9)
C35A—Ru2A—P2A—C20A	32.63 (13)	C35B—Ru2B—P2B—C13B	−160.80 (12)
C33A—Ru2A—P2A—C20A	−60.41 (12)	C34B—Ru2B—P2B—C13B	110.13 (12)
C34A—Ru2A—P2A—C20A	121.84 (12)	C33B—Ru2B—P2B—C13B	−67.64 (12)
Ru3A—Ru2A—P2A—C20A	−162.03 (9)	Ru1B—Ru2B—P2B—C13B	16.16 (10)
Ru1A—Ru2A—P2A—C20A	−141.87 (10)	Ru3B—Ru2B—P2B—C13B	38.09 (11)
C7A—P1A—C1A—C6A	135.0 (2)	C13B—P1B—C1B—C6B	−24.2 (2)
C13A—P1A—C1A—C6A	−118.4 (2)	C7B—P1B—C1B—C6B	−125.7 (2)
Ru1A—P1A—C1A—C6A	7.1 (2)	Ru1B—P1B—C1B—C6B	100.6 (2)
C7A—P1A—C1A—C2A	−49.2 (2)	C13B—P1B—C1B—C2B	159.3 (2)
C13A—P1A—C1A—C2A	57.4 (2)	C7B—P1B—C1B—C2B	57.9 (2)
Ru1A—P1A—C1A—C2A	−177.12 (19)	Ru1B—P1B—C1B—C2B	−75.9 (2)
C6A—C1A—C2A—C3A	1.9 (4)	C6B—C1B—C2B—C3B	3.0 (4)
P1A—C1A—C2A—C3A	−173.7 (2)	P1B—C1B—C2B—C3B	179.48 (19)
C6A—C1A—C2A—C26A	−175.1 (3)	C6B—C1B—C2B—C26B	−178.9 (3)
P1A—C1A—C2A—C26A	9.2 (4)	P1B—C1B—C2B—C26B	−2.4 (4)
C1A—C2A—C3A—C4A	0.2 (4)	C1B—C2B—C3B—C4B	−2.2 (4)
C26A—C2A—C3A—C4A	177.4 (3)	C26B—C2B—C3B—C4B	179.6 (3)
C2A—C3A—C4A—C5A	−2.0 (5)	C2B—C3B—C4B—C5B	0.0 (4)
C3A—C4A—C5A—C6A	1.7 (4)	C3B—C4B—C5B—C6B	1.4 (4)
C4A—C5A—C6A—C1A	0.5 (4)	C4B—C5B—C6B—C1B	−0.5 (4)
C2A—C1A—C6A—C5A	−2.3 (4)	C2B—C1B—C6B—C5B	−1.7 (4)
P1A—C1A—C6A—C5A	173.6 (2)	P1B—C1B—C6B—C5B	−178.2 (2)
C1A—P1A—C7A—C12A	135.6 (2)	C1B—P1B—C7B—C12B	−152.72 (19)
C13A—P1A—C7A—C12A	31.1 (2)	C13B—P1B—C7B—C12B	100.4 (2)
Ru1A—P1A—C7A—C12A	−93.2 (2)	Ru1B—P1B—C7B—C12B	−22.7 (2)
C1A—P1A—C7A—C8A	−48.8 (2)	C1B—P1B—C7B—C8B	36.6 (2)
C13A—P1A—C7A—C8A	−153.3 (2)	C13B—P1B—C7B—C8B	−70.3 (2)
Ru1A—P1A—C7A—C8A	82.4 (2)	Ru1B—P1B—C7B—C8B	166.63 (18)
C12A—C7A—C8A—C9A	−2.9 (4)	C12B—C7B—C8B—C9B	−0.7 (4)
P1A—C7A—C8A—C9A	−178.5 (2)	P1B—C7B—C8B—C9B	169.6 (2)
C12A—C7A—C8A—C27A	177.3 (3)	C12B—C7B—C8B—C27B	−179.9 (2)
P1A—C7A—C8A—C27A	1.7 (4)	P1B—C7B—C8B—C27B	−9.6 (4)
C7A—C8A—C9A—C10A	1.1 (4)	C7B—C8B—C9B—C10B	−0.9 (4)
C27A—C8A—C9A—C10A	−179.0 (3)	C27B—C8B—C9B—C10B	178.3 (3)
C8A—C9A—C10A—C11A	1.4 (4)	C8B—C9B—C10B—C11B	1.2 (4)
C9A—C10A—C11A—C12A	−2.2 (4)	C9B—C10B—C11B—C12B	0.2 (4)
C10A—C11A—C12A—C7A	0.4 (4)	C10B—C11B—C12B—C7B	−1.9 (4)
C8A—C7A—C12A—C11A	2.2 (4)	C8B—C7B—C12B—C11B	2.2 (4)
P1A—C7A—C12A—C11A	177.9 (2)	P1B—C7B—C12B—C11B	−169.1 (2)
C14A—P2A—C13A—P1A	−100.89 (16)	C1B—P1B—C13B—P2B	100.90 (16)
C20A—P2A—C13A—P1A	148.82 (15)	C7B—P1B—C13B—P2B	−149.05 (15)
Ru2A—P2A—C13A—P1A	22.80 (18)	Ru1B—P1B—C13B—P2B	−22.53 (18)
C7A—P1A—C13A—P2A	−125.87 (15)	C14B—P2B—C13B—P1B	−129.22 (15)
C1A—P1A—C13A—P2A	124.39 (15)	C20B—P2B—C13B—P1B	121.84 (15)
Ru1A—P1A—C13A—P2A	−3.25 (18)	Ru2B—P2B—C13B—P1B	0.44 (18)
C13A—P2A—C14A—C19A	23.7 (2)	C20B—P2B—C14B—C19B	−132.4 (2)
C20A—P2A—C14A—C19A	128.2 (2)	C13B—P2B—C14B—C19B	120.3 (2)
Ru2A—P2A—C14A—C19A	−100.5 (2)	Ru2B—P2B—C14B—C19B	−6.0 (2)
C13A—P2A—C14A—C15A	−160.4 (2)	C20B—P2B—C14B—C15B	48.4 (2)
C20A—P2A—C14A—C15A	−56.0 (2)	C13B—P2B—C14B—C15B	−58.9 (2)
Ru2A—P2A—C14A—C15A	75.3 (2)	Ru2B—P2B—C14B—C15B	174.80 (18)
C19A—C14A—C15A—C16A	−3.0 (4)	C19B—C14B—C15B—C16B	0.5 (4)
P2A—C14A—C15A—C16A	−178.8 (2)	P2B—C14B—C15B—C16B	179.7 (2)
C19A—C14A—C15A—C28A	178.8 (3)	C19B—C14B—C15B—C28B	−179.6 (3)
P2A—C14A—C15A—C28A	2.9 (4)	P2B—C14B—C15B—C28B	−0.4 (4)
C14A—C15A—C16A—C17A	1.6 (4)	C14B—C15B—C16B—C17B	−1.0 (5)
C28A—C15A—C16A—C17A	180.0 (3)	C28B—C15B—C16B—C17B	179.1 (3)
C15A—C16A—C17A—C18A	0.8 (5)	C15B—C16B—C17B—C18B	0.1 (5)
C16A—C17A—C18A—C19A	−1.9 (4)	C16B—C17B—C18B—C19B	1.3 (5)
C17A—C18A—C19A—C14A	0.5 (4)	C17B—C18B—C19B—C14B	−1.8 (5)
C15A—C14A—C19A—C18A	1.9 (4)	C15B—C14B—C19B—C18B	0.9 (4)
P2A—C14A—C19A—C18A	177.8 (2)	P2B—C14B—C19B—C18B	−178.4 (2)
C14A—P2A—C20A—C25A	143.5 (2)	C14B—P2B—C20B—C21B	51.6 (2)
C13A—P2A—C20A—C25A	−108.7 (2)	C13B—P2B—C20B—C21B	156.9 (2)
Ru2A—P2A—C20A—C25A	14.7 (2)	Ru2B—P2B—C20B—C21B	−79.6 (2)
C14A—P2A—C20A—C21A	−41.6 (3)	C14B—P2B—C20B—C25B	−134.1 (2)
C13A—P2A—C20A—C21A	66.3 (3)	C13B—P2B—C20B—C25B	−28.8 (2)
Ru2A—P2A—C20A—C21A	−170.3 (2)	Ru2B—P2B—C20B—C25B	94.7 (2)
C25A—C20A—C21A—C22A	−2.5 (5)	C25B—C20B—C21B—C22B	2.1 (4)
P2A—C20A—C21A—C22A	−177.4 (3)	P2B—C20B—C21B—C22B	176.54 (19)
C25A—C20A—C21A—C29A	178.0 (3)	C25B—C20B—C21B—C29B	−177.0 (2)
P2A—C20A—C21A—C29A	3.2 (5)	P2B—C20B—C21B—C29B	−2.7 (4)
C20A—C21A—C22A—C23A	2.9 (6)	C20B—C21B—C22B—C23B	−1.1 (4)
C29A—C21A—C22A—C23A	−177.7 (4)	C29B—C21B—C22B—C23B	178.1 (3)
C21A—C22A—C23A—C24A	−0.9 (6)	C21B—C22B—C23B—C24B	−0.4 (4)
C22A—C23A—C24A—C25A	−1.4 (5)	C22B—C23B—C24B—C25B	0.8 (4)
C21A—C20A—C25A—C24A	0.3 (4)	C23B—C24B—C25B—C20B	0.3 (4)
P2A—C20A—C25A—C24A	175.6 (2)	C21B—C20B—C25B—C24B	−1.8 (4)
C23A—C24A—C25A—C20A	1.7 (4)	P2B—C20B—C25B—C24B	−176.2 (2)

### Hydrogen-bond geometry (Å, °)

**Table:**

Cg1 is centroid of the C14A–C19A benzene ring.

**Table:**

*D*—H···*A*	*D*—H	H···*A*	*D*···*A*	*D*—H···*A*
C11B—H11B···O1B^i^	0.93	2.54	3.251 (3)	134
C24A—H24A···O5A^ii^	0.93	2.49	3.240 (4)	138
C29A—H29B···Cg1	0.96	2.96	3.702 (4)	135

Symmetry codes: (i) *x*, −*y*+1/2, *z*+1/2; (ii) *x*, −*y*+3/2, *z*+1/2.
